# Supplementary material for: A comparative systematic review and meta-analysis of uterine artery resistance in pregnant women with and without previous history of cesarean section
Source: PLoS One. 2025 Jun 18;20(6):e0325352. doi: 10.1371/journal.pone.0325352 (PMC12176234; doi:10.1371/journal.pone.0325352)
Supplement: S1 Table — (DOCX) [file pone.0325352.s001.docx]

Table S1: Detail of search strategies in each database and corresponding queries

| **database** | **query** | **N** |
| --- | --- | --- |
| PubMed | (Cesarean[tiab] OR Caesarean[tiab] OR “Deliver* Abdominal” [tiab] OR “Abdominal Deliver*”[tiab] OR Postcesarean[tiab]) AND Doppler[tiab] AND (“Uterine artery”[tiab] OR UtA[tiab]) | 205 |
| SCOPUS | TITLE-ABS (Cesarean OR Caesarean OR “Deliver* Abdominal” OR “Abdominal Deliver*” OR Postcesarean) AND TITLE-ABS (Doppler) AND TITLE-ABS ("Uterine artery" OR UtA) | 251 |
| EMBASE | (cesarean:ti,ab OR Caesarean:ti,ab OR ‘Deliver* Abdominal’:ti,ab OR ‘Abdominal Deliver*’:ti,ab OR Postcesarean:ti,ab) AND (doppler:ti,ab) AND ('uterine artery':ti,ab OR uta:ti,ab) | 280 |
| Web of science | TS=(cesarean OR Caesarean OR “Deliver* Abdominal” OR “Abdominal Deliver*” OR Postcesarean) AND TS=(doppler) AND TS=(“Uterine artery” OR UtA) | 222 |
