# Supplementary material for: A comparative systematic review and meta-analysis of uterine artery resistance in pregnant women with and without previous history of cesarean section
Source: PLoS One. 2025 Jun 18;20(6):e0325352. doi: 10.1371/journal.pone.0325352 (PMC12176234; doi:10.1371/journal.pone.0325352)
Supplement: S2 Table — (DOCX) [file pone.0325352.s002.docx]

Table S2: The evaluation of Certainty using the GradePro software and also Manually

| **Summary of findings:** | | | | | | |
| --- | --- | --- | --- | --- | --- | --- |
|  | | | | | | |
| **Patient or population:** Pregnant women undergoing second-trimester uterine artery Doppler ultrasound  **Setting:**  **Intervention:** History of cesarean section  **Comparison:** No prior cesarean section | | | | | | |
| Outcomes | **Anticipated absolute effects^*^** (95% CI) | | Relative effect (95% CI) | № of participants (studies) | Certainty of the evidence (GRADE) | Comments |
|  | **Risk with No prior cesarean section** | **Risk with History of cesarean section** |  |  |  |  |
| New outcome (PI) assessed with: Doppler ultrasound | - | SMD **0.15 SD more** (0.03 more to 0.26 more) | - | 1656 (6 non-randomised studies) | ⨁⨁◯◯ Low^a^ |  |
| ***The risk in the intervention group** (and its 95% confidence interval) is based on the assumed risk in the comparison group and the **relative effect** of the intervention (and its 95% CI).  **CI:** confidence interval; **SMD:** standardised mean difference | | | | | | |
| **GRADE Working Group grades of evidence** **High certainty:** we are very confident that the true effect lies close to that of the estimate of the effect. **Moderate certainty:** we are moderately confident in the effect estimate: the true effect is likely to be close to the estimate of the effect, but there is a possibility that it is substantially different. **Low certainty:** our confidence in the effect estimate is limited: the true effect may be substantially different from the estimate of the effect. **Very low certainty:** we have very little confidence in the effect estimate: the true effect is likely to be substantially different from the estimate of effect. | | | | | | |

| **Outcome** | **No. of Studies** | **Participants** | **Effect Estimate (95% CI)** | **Certainty (GRADE)** | **Notes** |
| --- | --- | --- | --- | --- | --- |
| **PI (Pulsatility Index)** | 6 | 1,656 | Hedges’s g = 0.15 [0.03, 0.26] | **Moderate** | Log-MoM transformation; consistent across studies; low heterogeneity |
