# Supplementary material for: A comparative systematic review and meta-analysis of uterine artery resistance in pregnant women with and without previous history of cesarean section
Source: PLoS One. 2025 Jun 18;20(6):e0325352. doi: 10.1371/journal.pone.0325352 (PMC12176234; doi:10.1371/journal.pone.0325352)
Supplement: S3 Table — (DOCX) [file pone.0325352.s003.docx]

Table S3: Results of Meta-Regression Analysis to Identify Potential Confounding Effects

| Meta-Regression Estimators | Coefficient | P>\|z\| | 95% Confidence Interval | |
| --- | --- | --- | --- | --- |
|  |  |  | Lower Bound | Upper Bound |
| Age*Group (C/S)Interaction | -0.11 | 0.10 | -0.25 | 0.02 |
| Age*Group (NVD)Interaction | -0.08 | 0.45 | -0.30 | 0.13 |
| BMI*Group (C/S)Interaction | 0.07 | 0.34 | -0.07 | 0.20 |
| BMI*Group (NVD)Interaction | 0.05 | 0.63 | -0.16 | 0.27 |
| DUS Week* Group(C/S)Interaction | -0.04 | 0.52 | -0.16 | 0.08 |
| DUS Week* Group(NVD)Interaction | -0.08 | 0.27 | -0.21 | 0.06 |
| Constant | 3.82 | 0.11 | -0.91 | 8.55 |

C/S Cesarean Section

NVD: Natural Vaginal Delivery

DUS : Doppler Ultrasonography

Residual heterogeneity:

tau2 = .02705

I2 (%) = 96.53

H2 = 28.86

R-squared (%) = 0.00

Wald chi2(6) = 3.22

Prob > chi2 = 0.7806
