# Supplementary figures and images for: A comparative systematic review and meta-analysis of uterine artery resistance in pregnant women with and without previous history of cesarean section
Source: PLoS One. 2025 Jun 18;20(6):e0325352. doi: 10.1371/journal.pone.0325352 (PMC12176234; doi:10.1371/journal.pone.0325352)

A

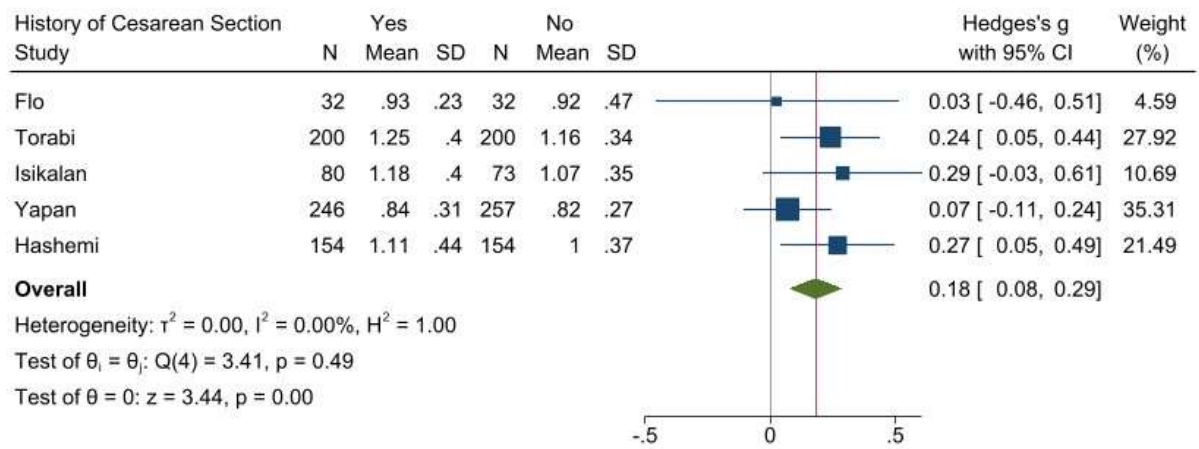

Random-effects DerSimonian-Laird model

B

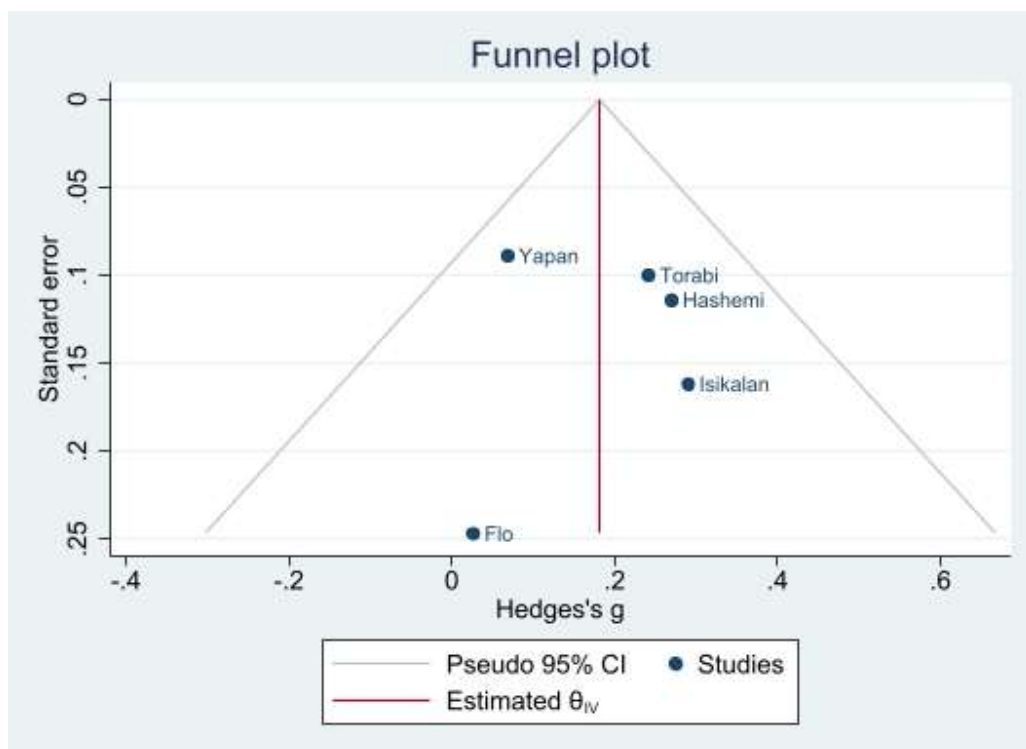

Supplement: S1 Fig — A: Forest plot of the PI meta-analysis following removal of the source of the heterogeneity. B: Funnel plot of the PI meta-analysis following removal of the source of the heterogeneity. (PDF) [file pone.0325352.s004.pdf]
