# Supplementary material for: A comparative systematic review and meta-analysis of uterine artery resistance in pregnant women with and without previous history of cesarean section
Source: PLoS One. 2025 Jun 18;20(6):e0325352. doi: 10.1371/journal.pone.0325352 (PMC12176234; doi:10.1371/journal.pone.0325352)
Supplement: Attachment — Submitted filename: Great job.docx [file pone.0325352.s005.docx]

**General Comments:**
The authors have done a commendable job in compiling and presenting the data. However, the manuscript would benefit from a thorough revision for grammar, sentence structure, and overall clarity to enhance readability and academic tone.

**Introduction:**

- Lines 50–51: Please provide a citation to support the statement.
- Line 57: Since multiple studies are referenced and no significant or negative outcomes are stated, more than one citation is needed to substantiate the claim.

**Methods:**

- Please define the abbreviations *PRISMA* and *PROSPERO* upon first use.
- Indicate the date on which the literature search was conducted.
- Line 78: Specify the version of the software which was used.
- When listing author contributions, include their initials in parentheses, e.g., (A.B. and C.D.).
- In the “Summary of Measures” section, clarify whether data extraction was performed by one or two researchers. Additionally, explain how discrepancies between reviewers were resolved.
- Since the results section includes a funnel plot, its use should also be described in the methods section.

**Results:**

- Clearly state the number of articles initially retrieved, how many were screened by title and abstract, how many underwent full-text review, and how many were ultimately included in the analysis.
- In the PRISMA flow diagram, one study is listed as coming from another source. Please clarify the origin of this study.

**Discussion:**

- Line 157: A citation is required to support the statement.
- The quality of evidence is typically evaluated using the GRADE tool. As your manuscript claims adherence to PRISMA guidelines, inclusion of GRADE assessment is recommended.
- Under limitations, it would be appropriate to mention the limited geographical generalizability of the findings, given that five studies were from Asia and one from Europe.
- Consider including the strengths of your study alongside the limitations.
- It would be beneficial to include recommendations for future research directions in the conclusion section.

**Table 1:**

- In Row 2, the reference appears in the middle of the author’s name. Please correct this.

**Figure 1:**

- Indicate how many results were retrieved from each database.
- Specify the reasons for exclusion and the number of studies excluded for each reason.
