# Supplementary material for: A comparative systematic review and meta-analysis of uterine artery resistance in pregnant women with and without previous history of cesarean section
Source: PLoS One. 2025 Jun 18;20(6):e0325352. doi: 10.1371/journal.pone.0325352 (PMC12176234; doi:10.1371/journal.pone.0325352)
Supplement: Attachment — Submitted filename: In Line 72.docx [file pone.0325352.s007.docx]

In Line 72, the manuscript mischaracterizes the PECO model as being based on interventions; however, PECO is more appropriately used for observational studies and is structured around exposure rather than intervention. This should be corrected, and an appropriate citation supporting the use of the PECO framework should be added.

The study question is presented using the PECO format, but the individual components—Population, Exposure, Comparator, and Outcome—are not clearly defined. Each element should be explicitly stated to ensure clarity and methodological transparency.

The manuscript refers to the PRISMA guideline but does not provide a proper citation. A formal reference to the most current version of the PRISMA guideline (e.g., PRISMA 2020) should be included.

The phrase “including papers, published and unpublished thesis” is imprecise. It should be revised to clearly describe the types of literature included in the review, specifying the nature of the unpublished materials and the sources used to retrieve them.

The response to the earlier comment regarding the timing of the literature search is insufficient. The manuscript must explicitly indicate the date on which the literature search was conducted by your team for each database or platform. Merely referencing Line 83 is inadequate; the information should be clearly presented in the methods section.

As noted previously, the flow diagram in Figure 1 lacks specificity regarding exclusion criteria. The reasons for exclusion should be clearly stated both at the title and abstract screening stage and at the full-text review stage, in line with best practices for systematic review reporting.
